# Unexpected catalytic activity of nanorippled graphene

**DOI:** 10.1073/pnas.2300481120

**Published:** 2023-03-13

**Authors:** P. Z. Sun, W. Q. Xiong, A. Bera, I. Timokhin, Z. F. Wu, A. Mishchenko, M. C. Sellers, B. L. Liu, H. M. Cheng, E. Janzen, J. H. Edgar, I. V. Grigorieva, S. J. Yuan, A. K. Geim

**Affiliations:** ^a^Department of Physics and Astronomy, University of Manchester, Manchester M13 9PL, UK; ^b^National Graphene Institute, University of Manchester, Manchester M13 9PL, UK; ^c^Key Laboratory of Artificial Micro- and Nano- structures of Ministry of Education, School of Physics and Technology, Wuhan University, Wuhan 430072, China; ^d^Shenzhen Graphene Center, Institute of Materials Research, Tsinghua Shenzhen International Graduate School, Tsinghua University, Shenzhen 518055, China; ^e^Tim Taylor Department of Chemical Engineering, Kansas State University, Manhattan, KS 66506

**Keywords:** graphene, 2D materials, catalysis, hydrogen dissociation, proton transport

## Abstract

Graphite is one of the most chemically inert materials. Its elementary constituent, monolayer graphene, is generally expected to inherit most of the parent material’s properties including chemical inertness. Here, we show that, unlike graphite, defect-free monolayer graphene exhibits a strong activity with respect to splitting molecular hydrogen, which is comparable to that of metallic and other known catalysts for this reaction. We attribute the unexpected catalytic activity to surface corrugations (nanoscale ripples), a conclusion supported by theory. Nanoripples are likely to play a role in other chemical reactions involving graphene and, because nanorippling is inherent to atomically thin crystals, can be important for two-dimensional (2D) materials in general.

## Significance

Graphene, an isolated atomic plane of graphite, is generally expected to inherit most of graphite’s properties. These expectations are reported to be wrong as far as chemical activity of the two materials is concerned. Indeed, graphite is one of the most inert materials known in nature. In contrast, graphene is shown here to dissociate molecular hydrogen as strongly as the best catalysts known for this reaction. This is attributed to the fact that graphene monolayers are not flat (as within graphite) but unavoidably have nanoscale ripples that serve as active sites for hydrogen splitting. The results have implications for all two-dimensional (2D) materials that being inherently nonflat may exhibit chemical and catalytic properties very different from their bulk counterparts.

Despite inheriting chemical inertness from graphite and being highly stable in air, graphene has widely (and perhaps somewhat surprisingly) been considered for the use in catalysis (1–7). The primary reason is the huge surface area of graphene such that it can act as an efficient support and delivery platform for dispersed catalytically active nanoparticles and functional groups (1–5). Another reason is that graphene itself can be modified relatively easily to provide a high density of vacancies, substitutional dopants, edges, and other atomic-scale defects, which can potentially provide catalytically active sites (1–7). However, it was also noticed occasionally that even defect-free graphene monocrystals (obtained by mechanical exfoliations) could be more chemically reactive than bulk graphite. For example, monolayer graphene reacts with oxygen more easily than few-layer graphene and graphite (8). Also, wrinkles and strained regions of defect-free graphene were reported to accelerate its functionalization with certain chemicals (9–13). Despite those few observations, it remains unknown if pristine graphene (defect-free monolayers without wrinkles and macroscopic strain) is chemically inert similar to graphite. One recent finding indicates that this might not be the case (14). Indeed, defect-free graphene membranes allow discernable permeation of molecular hydrogen, despite being completely impermeable to smaller and generally more permeating helium atoms (14). To explain this conundrum, it was speculated that, unlike graphite, graphene could split molecular hydrogen into two protons (hydrogen atoms adsorbed on graphene), and then, those subatomic particles permeated through the graphene lattice, a feat essentially impossible for any gas under ambient conditions (15, 16). Although rather convoluted, this explanation was supported by theoretical predictions that strongly curved regions of graphene could dissociate molecular hydrogen (17–19) and the experimental fact that nominally flat two-dimensional (2D) membranes exhibit ubiquitous nanoscale ripples with substantial strain and curvature (20–23).

In this report, we provide clear evidence for hydrogen dissociation on pristine graphene using three complementary sets of experiments. First, we have compared hydrogen permeation through graphene with that through the so-called “white graphene”, monolayers of hexagonal boron nitride (hBN). This comparison is critical because the two 2D materials are structurally similar but have different electronic spectra which makes hydrogen dissociation possible only on graphene’s nanoripples, according to theory (18, 19). No sign of hydrogen permeation through hBN monolayers could be found experimentally, despite the latter being hundred times more proton conductive than graphene (15, 16). The provided side-by-side comparison between these two rather similar monolayer materials also rules out any possible artifacts (e.g., influence of accidental defects). Second, we have used the Raman spectroscopy to monitor the development of the D peak that appears if molecular hydrogen is split into atoms and those bind to the carbon lattice (24, 25). We find that the D peak appears for graphene placed onto a silicon oxide substrate that is relatively rough and allows nanoripples, whereas no sign of hydrogen adsorption could be detected for either ripple-free (atomically flat) graphene or bulk graphite’s surface. These observations corroborate that roughness plays a critical role in hydrogen dissociation and emphasize further the difference between reactivities of defect-free graphene and graphite surfaces. Third, using a mixture of hydrogen (H_2_) and deuterium (D_2_) gases, we show that graphene acts as a powerful catalyst converting H_2_ and D_2_ into hydrogen deuteride (HD) in contrast to graphite and other carbon-based materials under the same conditions. Graphene’s catalytic efficiency per gram exceeds by far that of traditional catalysts for the reaction.

## Results

### Hydrogen Permeation through Graphene and hBN Monolayers.

**Fig. 1.**
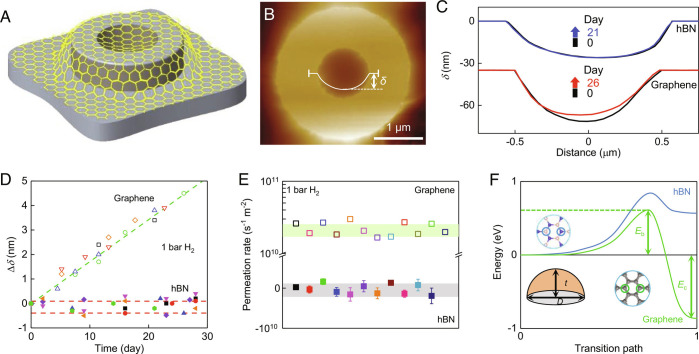
Hydrogen transport through graphene and hBN membranes. (*A*) Schematic of the microcontainers. (*B*) AFM image of one of our microcontainers sealed with an hBN monolayer. The white curve shows the height profile along the membrane center. (*C*) Height profiles for two containers sealed with graphene and hBN before and after their storage in hydrogen for a few weeks (color-coded curves). *T* = 295 ± 3 K; *P* = 1 bar. Note the different scales for the *x*- and *y*-axes, which serve to show changes in *δ* and exaggerate the inward curving of 2D membranes. This sagging is caused by graphene’s adhesion to inner walls of the microcontainers. The macroscopic curvature due to sagging was less than 4% and insufficient to cause hydrogen dissociation, according to theory (18, 19). Nanoscale rippling provides considerably higher curvature and strain (20, 22, 23). (*D*) Changes in the height Δ*δ* for hBN (solid symbols) and graphene (open) membranes measured over one month. Each symbol in (*D* and *E*) denotes a different microcontainer with more than 10 tested for each material. The red dashed lines outline the full range of Δ*δ* for the hBN membrane. Green line: Best linear fit for the graphene data shown by the green symbols. (*E*) Permeation rates evaluated from the Δ*δ* measurements. Note a break in the y-axis in (*E*): The scale is linear below 10^10^ and logarithmic above. Error bars: SD for the linear fits of Δ*δ* as a function of time in (*D*) and shown only if larger than the symbols. Shaded areas: overall SD for the graphene and hBN measurements. (*F*) Energy profiles for dissociation of H_2_ on graphene and hBN ripples for the case of two hydrogen atoms being adsorbed at the central positions as shown schematically in the *Insets*. Other adsorption positions are less favorable for hydrogen splitting as discussed in *SI Appendix*. The curvature *t/D* (*Low*-*Left* *Inset*) is 12% for both curves in (*F*).

We used the same setup and procedures as described in detail previously (14). Briefly, micrometer-size containers were fabricated from monocrystals of graphite and sealed with monolayers of either graphene or hBN (Fig. 1 *A* and *B*). The microcontainers were placed in a gas chamber filled with, e.g., helium. If defects were present or the sealing was imperfect, the membranes started bulging because of a gradually increasing pressure inside (*SI Appendix*, Fig. S1). The bulging could be monitored by atomic force microscopy (AFM), and changes in the membrane position *δ* with time (Fig. 1 *B* and *C*) could be translated directly into permeation rates (14, 26). This technique is remarkably sensitive, allowing detection of even a single vacancy if present in graphene membranes (26). In the absence of defects, our microcontainers were completely impermeable to any inert gas within an experimental accuracy of a few atoms per hour (leak rate < 10^9^ s^−1^ m^−2^) (14).

We placed such helium-proof microcontainers inside a chamber filled with hydrogen under ambient conditions and found that graphene membranes slowly but steadily bulged out over days (Fig. 1 *C* and *D*). The measured leak rate of ~2 × 10^10^ s^−1^ m^−2^ (Fig. 1 *D* and *E*) agreed with our earlier report (14). As the energy barrier for helium permeation through graphene is predicted to be several electron volts and even higher for molecular hydrogen, the only possibility for hydrogen to pass through defect-free graphene is as protons because the latter face a relatively low barrier of about 1 eV (for the case of graphene monolayers) (16). Accordingly, the hydrogen gas permeation has been explained by a two-stage mechanism: First, molecular hydrogen dissociates on graphene (on top of nanoripples as suggested by theory) (14, 18, 19), and then, the resulting hydrogen adatoms, indistinguishable from adsorbed protons, flip to the other side of graphene by overcoming the same barrier as measured for proton transport (15, 16). This part of our report repeats the earlier experiments and is provided here only to make a clear comparison between hBN and graphene monolayers (see below).

In stark contrast to graphene, similar containers but sealed with hBN monolayers did not bulge in either helium or hydrogen atmosphere (that is, exhibited no sign of hydrogen permeation; Fig. 1 *C* and *D*). If hBN were to dissociate hydrogen (the first stage of the above mechanism), then hydrogen would permeate much more quickly through hBN than graphene because the former is two orders of magnitude more proton transparent (15). The absence of any discernable hydrogen permeation proves that, unlike graphene, hBN is not active for splitting H_2_, in agreement with our density functional theory calculations (*SI Appendix*, Figs. S2 to S5).

These calculations show a clear difference between graphene and hBN. If their monolayers are flat, the dissociation barrier *E*_b_ (calculated as the maximum energy at the transition state, as illustrated in Fig. 1*F*) is very high (>3 eV) for both cases. Ripples with considerable curvature *t/D* can reduce the barrier to less than 1 eV (where *t* is the height of a ripple and *D* its lateral dimension; *Inset* of Fig. 1*F*). However, hydrogen dissociation can be energetically favorable only for graphene (that is, it becomes an exothermic reaction characterized by the chemisorption energy *E*_c_ <0, as shown in Fig. 1*F*). This requires curvatures above ~10% (Fig. 1*F* and *SI Appendix*, Fig. S2). Such strongly curved nanoripples are routine for graphene membranes. Their rippling was extensively studied by transmission electron microscopy (20) and scanning tunneling microscopy (22, 23) and attributed to both local strain and thermal fluctuations (20, 21, 23). In the case of hBN monolayers, hydrogen dissociation is calculated to remain energetically unfavorable (*E*_c_ > 0) for all realistic curvatures and strains (<15%) that can be supported by 2D crystals without breaking (Fig. 1*F* and *SI Appendix*, Figs. S2–S4).

The above results suggest a dramatic difference in chemical and catalytic activity of graphene and hBN monolayers, at least with respect to hydrogen. However, with only hundred molecules per hour permeating inside graphene microcontainers (14), neither experiment nor theory provides any indication whether graphene is a good or bad catalyst for hydrogen dissociation.

### Role of Graphene’s Nonflatness.

The described mechanism of hydrogen dissociation by graphene nanoripples implies that, if placed in a hydrogen atmosphere, graphene’s surface should contain a certain amount of adsorbed hydrogen atoms. Their binding to carbon atoms of the crystal lattice (C-H bonds) is strong (sp^3^ type) (18, 19), especially if hydrogen atoms occupy neighboring carbon sites, like in the case of a fully hydrogenated graphene derivative, graphane (24, 25). Such sp^3^ adatoms should give rise to the D peak in graphene’s Raman spectra. Accordingly, this peak can be used as a measure of graphene’s reactivity with respect to hydrogen dissociation.

**Fig. 2.**
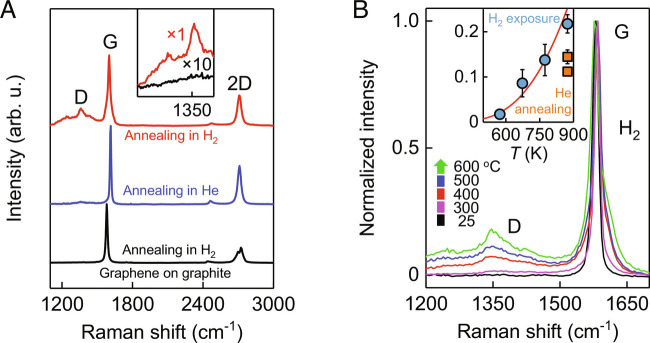
Raman spectroscopy of graphene exposed to hydrogen. (*A*) Raman spectra of monolayer graphene after its exposure to 1-bar hydrogen at 600 °C for 2 h (red curve). Blue curve: same but for helium. Black curve: same measurements but for atomically flat graphene placed on graphite without alignment and exposed to hydrogen at 600 °C for 4 h. The spectra were taken at room *T* and, for clarity, are shifted vertically. All spectroscopy parameters were exactly the same: wavelength, 514.5 nm; power, ~1.7 mW; spot size, ~2 μm; acquisition time for each curve, 1 h. The *Inset* magnifies the D peak region on the red and black curves with the intensity for the black curve being amplified 10 times. (*B*) Raman spectra of graphene in hydrogen taken in situ with increasing *T* (color coded). *Inset*: *T* dependence of the D peak intensity relative to that of the G peak (circles). Squares: hydrogen-induced D peak decreases after 4 and 8 h of annealing at 600 °C in a helium atmosphere (top and bottom squares, respectively). The spectra were taken from the same spots away from graphene edges. Error bars: SD for at least three different spots used in the Raman measurements. Solid curve: guide to the eye showing the Arrhenius dependence with an activation energy of 0.4 eV.

At room temperature (*T*), the coverage of graphene with hydrogen adatoms is expected to be extremely sparse (~100 hydrogen molecules permeate through micrometer-scale membranes during an entire hour) (14), and indeed, we did not observe the appearance of any discernable D peak for monolayer graphene placed in a hydrogen atmosphere for many days. To increase the coverage, graphene can be heated to help overcome a finite energy barrier required for hydrogen dissociation (Fig. 1*F*). Moreover, thermal fluctuations at higher *T* can also generate dynamic nanoripples with high curvature (21, 23). With these considerations in mind, we heated graphene monolayers to a certain *T* in pure H_2_ and compared their Raman spectra with similarly treated graphene but in He. For these measurements, graphene was exfoliated directly onto a silicon oxide wafer because suspended graphene cannot withstand high *T* and breaks down, presumably due to induced mechanical strain (14). Note that the silicon oxide surface supporting graphene was relatively rough with a rms roughness of ~0.5 nm (*SI Appendix*, Fig. S6). As shown in Fig. 2*A*, graphene crystals heat-treated in hydrogen and then cooled down to room *T* exhibited a pronounced D peak, indicating the appearance of numerous sp^3^ defects. In contrast, similar graphene samples but heated in a helium atmosphere showed no sign of the D peak, proving qualitatively that graphene could react only with hydrogen.

Next, we used the D peak to assess a role of graphene’s surface roughness in the hydrogenation process. To this end, the above Raman results were compared with those obtained for similarly exfoliated graphene monolayers but placed onto an atomically flat surface of graphite (*SI Appendix*, Fig. S6). After exposing the flat graphene to 600 °C in pure H_2_ for several hours, no sign of the D peak could be discerned in the Raman spectra, in clear contrast to the case of nonflat graphene (Fig. 2*A*). Neighboring graphite surfaces also showed no D peak. Note that, despite different thicknesses, graphene monolayers and bulk graphite exhibit similar intensities of the G peak (Fig. 2*A*), in agreement with the previous studies (27). Accordingly, if the D peak even with a 100 times smaller intensity were present for graphene placed on graphite, our sensitivity would certainly allow its detection (*Inset* of Fig. 2*A*). In another control experiment, we prepared graphene with intentionally introduced sp^3^ defects (e.g., tears) and again placed the samples on atomically flat graphite. In this case, the D peak was clearly seen, proving that we would easily see hydrogen adatoms if they were also adsorbed on flat graphene.

Consecutive annealing of hydrogenated (nonflat) graphene in He or vacuum reduced the D peak, although a notable hump remained in the relevant spectral region even after several days at 600 °C (*Inset* of Fig. 2*B* and *SI Appendix*, Fig. S7). This hump is likely to be due to small regions of thermally stable graphane, which is formed if graphene is covered with atomic hydrogen on both sides (24). To quantify the observed hydrogenation process, Fig. 2*B* shows consecutive Raman spectra taken inside a 1-bar hydrogen atmosphere with increasing *T*. The D peak gradually grows with increasing *T*, and this dependence allows an estimate for the hydrogen reaction activation energy as ~0.4 eV.

The results reported in this section suggest that nonflatness is essential for graphene’s reactivity and that the activation energy for hydrogen dissociation is relatively small. However, they still provide no indication about graphene’s efficiency as a catalyst. The latter is assessed in the next section.

### Enhanced Hydrogen Isotope Exchange.

Annealing hydrogenated graphene in helium or vacuum shows that hydrogen adatoms desorb from graphene’s surface (*Inset* of Fig. 2*B*) and then presumably recombine into molecular hydrogen. This agrees with the proposed mechanism of hydrogen gas permeation through graphene membranes, which involves recombination of permeating protons (atomic hydrogen) inside microcontainers (14–16). If instead of pure H_2_, graphene is exposed to a mixture of H_2_ and D_2_, one should expect hydrogen and deuterium adatoms to recombine at random, forming an HD gas (in addition to H_2_ and D_2_). Therefore, detection of HD would provide an unequivocal proof for hydrogen dissociation on graphene, and using this reaction, graphene could also be benchmarked against known catalysts.

**Fig. 3.**
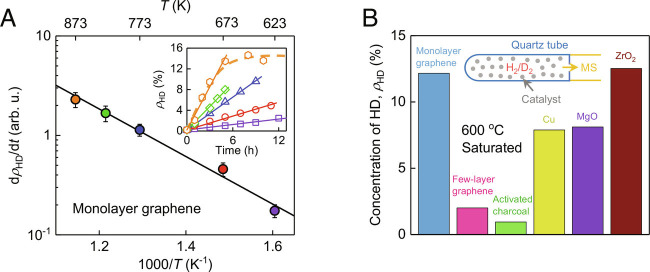
Hydrogen–deuterium exchange catalyzed by graphene. (*A*) Temperature dependence of HD production rates for a monolayer graphene powder. Symbols: experimental data at different *T* with error bars indicating SD. Solid curve: best exponential fit, yielding the activation energy of ~0.4 eV. *Inset*: *ρ*_HD_ as a function of time *t* at different *T* (color coding for *T* as in the main panel). Solid lines: linear fits for the initial stage of HD production. Dashed curve: guide to the eye. Our accuracy of determining the HD concentrations was ±2% as found in repeated measurements under the same conditions. (*B*) Final concentration of HD after annealing the H_2_–D_2_ mixtures using various catalysts and reaching saturation in *ρ*_HD_ as a function of time (5 h at 600 °C in all cases). *Inset*: Schematic of the experimental setup where MS refers to “mass spectrometer”.

Because individual graphene crystals generate such a minute amount of HD, it is impossible to detect it by mass spectrometry. Accordingly, we chose to use a graphene powder for these experiments. It was obtained through careful reduction of graphene oxide, which induced little defects in the basal plane (*ACS Material*; *SI Appendix*, Fig. S8). The powder was well characterized and contained highly corrugated and mostly isolated (nonrestacked) monolayers with the measured specific surface area of ~1,000 m^2^ g^−1^ (28). A quartz tube was fully filled with this powder, and a mixture of hydrogen and deuterium gases (*P*_H2_ = *P*_D2_ ≈ 0.5 bar) was then added (*SI Appendix*). After a certain exposure time, the mixed gas was analyzed by mass spectroscopy to determine the resulting HD concentration, *ρ*_HD_. At room *T*, we could not detect any HD either in the presence of graphene or without it (within our experimental accuracy, better than 0.2%, being limited by the background HD present inside the commercially supplied D_2_). To accelerate the reaction of H_2_ with D_2_, the gas mixture was heated up. At 600 °C, the monolayer graphene powder led to formation of HD, and its concentration increased as a function of time saturating at ~15% after approximately 5 h (*Inset* of Fig. 3*A*). No HD could be detected in the absence of graphene. The dissociation–recombination reaction H_2_ + D_2_ ↔ 2HD can result in *ρ*_HD_ up to 50% if H_2_ and D_2_ molecules are fully split and then recombined stochastically into HD, H_2_, and D_2_ products. The observed saturation below 50% can be attributed to “poisoning”, a standard occurrence for catalysts (Fig. 3*B* and *SI Appendix*, Fig. S9). For graphene, the poisoning is probably caused by formation of chemically inert regions of graphane (24, 25), a conclusion also supported by the Raman behavior discussed in the previous section. Furthermore, the *Inset* of Fig. 3*A* shows that during initial stages, the HD reaction proceeded at constant production rates, d*ρ*_HD_/d*t*. By plotting these rates as a function of *T*, we have found that they follow the Arrhenius behavior (Fig. 3*A*). The fit yields the activation energy of ~0.4 eV, which agrees well with our estimate obtained from the Raman spectroscopy (Fig. 2*B*).

As a reference, we carried out the same measurements using known catalysts for the HD reaction, namely, zirconia, MgO, and Cu (29, 30). They were also in the powder form as detailed in *SI Appendix*. The HD production for these catalysts exhibited time dependences qualitatively similar to that of the monolayer graphene powder (*SI Appendix*, Fig. S9). The saturation in *ρ*_HD_ at 600 °C occurred after a few hours and was below 50%, indicating poisoning of the reference catalysts (Fig. 3*B*). Next, to assess a possible role played in the production of HD by various atomic-scale defects in graphene such as vacancies and edges as well as by residual oxygen and other functional groups present in the graphene powder (28), we have compared it with other possible carbon-based catalysts (namely, few-layer graphene and activated charcoal). Both showed substantially lower catalytic activity for the exchange reaction (Fig. 3*B*). Our few-layer graphene used in the control experiments was a powder obtained by ultrasonic exfoliation (31). Its flakes were similar in size to those in the monolayer graphene powder (several micrometers) but considerably thicker (typically, 10 layers). They were notably less corrugated (*SI Appendix*, Fig. S8), as expected (20–23). Few-layer graphene showed much less efficiency than monolayer graphene in terms of HD production not only per g but also per surface area (~30 times less efficient per m^2^; see *SI Appendix*, Fig. S9*D*). This agrees well with the fact that few-layer graphene has a much less rippled surface than monolayer graphene. Furthermore, activated charcoal known for its highly porous structure provided numerous broken carbon bonds and oxygen surface groups (32). Nonetheless, its catalytic efficiency for HD production was only minor, confirming that defects (possibly present in minor amounts) play a little role in the observed hydrogen dissociation by graphene. The above comparison between different graphitic materials corroborates that nanoripples—which are unavoidable for graphene monolayers, less profound for few-layer graphene, and absent in charcoal—play an important role in the observed catalytic activity.

To compare catalytic efficiency of the tested materials more quantitatively, we also translated their *ρ*_HD_ at saturation (Fig. 3*B*) into the number of HD molecules, *N*_HD_, produced per gram or surface area of the catalysts. *SI Appendix*, Fig. S9*C* shows that, in terms of the weight catalytic efficiency (*N*_HD_ per g), graphene excels all the other powders by orders of magnitude. This is helped by the fact that graphene is essentially a surface and has no bulk (theoretical surface area of ~2,600 m^2^ g^−1^), whereas the other powders have their bulk mostly inaccessible for the HD production. Even in terms of the surface efficiency (*N*_HD_ per m^2^), graphene compares favorably with MgO and ZrO_2_ and is surpassed only by Cu (*SI Appendix*, Fig. S9*D*). From the known *N*_HD_, we can estimate the turnover number *TON* defined as the maximum yield attainable from a catalytic center (33). Assuming that all carbon atoms of graphene contribute equally to the reactivity, we find *TON* ≈ 0.1. On the other hand, the D peak intensity in the Raman spectra of graphene in Fig. 2*A* indicates (24) that only 8 to 10% of graphene’s atoms took part in the reaction before the material got poisoned. Those were presumably carbon atoms located in the most curved part of graphene where the activation energy was strongly reduced (Fig. 1*F*). Accounting only for the latter atoms as catalytic centers, we obtain *TON* ≈ 1. Finally, if we consider individual nanoripples as catalytic centers, this yields huge *TON* ≈ 10^3^ to 10^4^. Note that, from the perspective of traditional catalysis, these estimates have only a limited sense because of the difficulty with defining what catalytic centers are in our case. In particular, carbon atoms on dynamic ripples may change in time from being reactive to nonreactive and back. However, even our most conservative estimate of *TON* ≈ 1 makes graphene competitive with traditional catalysts because in the latter case, only near-surface atoms play a role in stimulating reactions, whereas most of graphene’s atoms partake in nanorippling and thus are reactive.

## Discussion

A flat sheet of graphene is expected to be highly stable and chemically inert under ambient conditions similar to its parent material, graphite. However, graphene monolayers are never perfectly flat because of thermal fluctuations (flexural phonons) and practically unavoidable local strain, which generate static nanoscale wrinkles and ripples (20–23). Our experiments show that in terms of reactivity, such nanorippled graphene is quite different from both graphite and atomically flat graphene. This offers strong support for earlier theoretical studies of hydrogen dissociation on curved graphene (14, 17–19) and, also, indicates that nanoripples can be more important for catalysis than the “usual suspects” such as, for example, vacancies, edges, and residual functional groups on graphene’s surfaces. To enhance graphene’s catalytic activity, one can increase the density of nanoripples by increasing *T* that induces thermal fluctuations and creates dynamic ripples or by placing graphene onto rough substrates (static ripples). Furthermore, additional ripples can be formed by thermal cycling of graphene on various substrates due to different thermal expansion coefficients. Because nanorippling is inherent for all atomically thin crystals, it is worth keeping in mind that the enhancement of chemical and/or catalytic activity is also possible for the case of other 2D materials and for other chemical reactions. For example, bulk MoS_2_ and other chalcogenides are often used as three-dimensional (3D) catalysts but may exhibit even stronger activity in the 2D form.

Our results have implications for many previous observations reported in the literature. For example, nanoripples can be relevant for graphene oxidation that occurs preferentially on monolayers and wrinkles but has remained unexplained (8, 11). To this end, note that monolayer hBN (no activity with respect to hydrogen splitting as found in our work) also exhibits much stronger thermal oxidation resistance than graphene (34, 35). This is consistent with their different electronic spectra that are ultimately responsible for the reported difference in hydrogen dissociation on nanoripples. Our results also support the idea of hydrogen storage inside carbon nanotubes (17). Indeed, molecular hydrogen can adsorb and dissociate on strongly curved graphene surfaces, then flip through as protons, and finally desorb from the inner concave surface so that H_2_ can potentially be stored inside carbon nanotubes. Furthermore, in heterogeneous catalytic reactions involving, for example, graphene-coated metal surfaces and nanoparticles (36–40), the local curvature of graphene can potentially account for some enhanced reactivity, a mechanism disregarded so far. The hydrogen dissociation on nanoripples may also play a role in other reactions involving graphene-based catalysts (e.g., in electrolysis and electrocatalysis) (12, 36–40).

## Supplementary Material

Appendix 01 (PDF)

## Data Availability

All study data are included in the article and/or *SI Appendix*.
