# Supplementary material for: Unexpected catalytic activity of nanorippled graphene
Source: Proc Natl Acad Sci U S A. 2023 Mar 13;120(12):e2300481120. doi: 10.1073/pnas.2300481120 (PMC10041176; doi:10.1073/pnas.2300481120)
Supplement: Supplementary file 1 — Appendix 01 (PDF) [file pnas.2300481120.sapp.pdf]

Supporting Information for

Unexpected catalytic activity of nanorippled graphene.

P. Z. Sun, W. Q. Xiong, A. Bera, I. Timokhin, Z. F. Wu, A. Mishchenko, M. C. Sellers, B. L. Liu, H. M. Cheng, E. Janzen, J. H. Edgar, I. V. Grigorieva, S. J. Yuan, A. K. Geim

Corresponding authors

Andre K. Geim
Email: geim@manchester.ac.uk
Pengzhan Sun
Email: pengzhansun@um.edu.mo

This PDF file includes:

Supporting information text
Figures S1 to S9
SI References

Supporting Information Text

Materials and methods

1. Microcontainers for hydrogen permeation experiments. To fabricate such containers, we followed the procedures developed in ref. (1). Monocrystals of graphite with a thickness of 150–200 nm were mechanically exfoliated onto an oxidized silicon wafer. The crystals' surface was carefully examined in an optical microscope using both dark-field and differential-interference-contrast modes. Areas free from contamination and atomic terraces were selected for the next step that involved electron-beam lithography. It was employed to make a polymer mask defining an array of rings with inner diameters of 0.5–1 μm and rims having a width of ~1 μm. Dry etching was used to project the mask geometry into the graphite crystals, which created microwells having ~80 nm depth (1). After dissolving the polymer mask, the structures were annealed at 400 °C in a H_2/Ar atmosphere for several hours to remove polymer residues. Next, large (> 100 μm in size) graphene or hBN monolayers were obtained by mechanical exfoliation and transferred on top of the microwells to seal them, creating microcontainers (Fig. 1*B*).

The resulting microcontainers were first inspected by AFM (*Fastscan* from *Bruker*) for any damage in the atomically tight sealing (1) and possible defects in suspended membranes (e.g., tears, cracks and wrinkles). Only microcontainers without discernible imperfections progressed to the next stage that was leakage tests. First, microcontainers were placed inside a stainless-steel chamber that was pressurized with a heavy inert gas (for example, Ar or Xe under a typical pressure of 3 bar). After being stored for one week, the microcontainers were quickly taken out and, within a few minutes, measured by AFM to find possible changes in the membrane position δ (Figs. 1*B*, *C*). Such changes would indicate the presence of atomic-scale defects allowing gas permeation inside microcontainers (1-4). Only those exhibiting no changes in δ (within our experimental accuracy of better than 1 nm; Fig. 1*D*) were further tested by placing them into a 1-bar helium atmosphere for one month. This test ensured that even smallest defects such as individual vacancies were absent (2), that is, the microcontainers were perfectly sealed and defect free (1). These helium-tight devices were used in the hydrogen permeation measurements described in the main text.

2. Leak tests using silicon-oxide microcontainers. The above tests could easily detect atomic-scale defects (2) but thousand times larger defects that allowed very rapid deflation of microcontainers (quicker than within a few minutes) could not be revealed using the described approach. Indeed, no changes in δ would be detected not only for He-tight microcontainers but also those with large cracks in membranes. To rule out the latter possibility, we mostly relied on dedicated atomic force and scanning electron microscopy (1, 2). Nonetheless, as an additional proof that large defects were generally absent in our graphene and hBN membranes, we tested microcontainers made from oxidized silicon wafers, as first explored in refs. (3, 4). The difference is that amorphous SiO_2 does not provide good sealing but allows a slow gas permeation through it (3, 4), unlike our monocrystalline containers with atomically-tight sealing (1). Therefore, if one compares similarly made and sealed silicon-oxide and monocrystalline microcontainers, only the former should exhibit notable bulging in Ar and He (3, 4).

With this test in mind, we etched microwells in an oxidized silicon wafer (300 nm of SiO_2) and sealed them with exfoliated graphene or hBN monolayers (Fig. S1*A*). The microcontainers were typically ~3 μm in diameter and ~200 nm deep. Permeation of various inert gases into such microcontainers was measured following the procedures described above and in refs. (1-4). Taking monolayer hBN and Ar as an example in Fig. S1*B*, the suspended membranes clearly bulged out after their pressurization. Their consecutive deflation in air was monitored by AFM as a function of time, yielding a deflation rate of ~0.7 nm/h (Fig. S1*B*). This translates into a permeance of ~2×10^{-27} mol s^{-1} Pa^{-1} for Ar, which is consistent with the previously reported values for SiO_2 microcontainers (3, 4). Similar agreement was also observed for other gases including He. This corroborates the previous conclusions that the leakage occurs through SiO_2 rather than monocrystalline membranes. Our extensive leakage tests unambiguously prove that the membranes made from exfoliated graphene and hBN monolayers were completely free from either

microscopic or macroscopic defects. Accidental large defects were ruled out by AFM and electron microscopy.

3. Density functional theory (DFT) calculations. Dissociation of molecular hydrogen on graphene and hBN nanoripples was simulated using DFT, as implemented in Vienna *ab initio* package (5). The ion-electron interactions and exchange correlation potential were described using the projected augmented wave and generalized gradient approximation (6). The kinetic energy cutoff and *k*-point meshes were set at 500 eV and 3×3×1, respectively (7). To avoid periodic interactions, a vacuum region of 20 Å was adopted. The convergences for the stress force and total energy were set as 0.02 eV/Å and 10^{-5} eV, respectively. The van der Waals interactions of H_2 with graphene and hBN were treated by the semi-empirical DFT-D3 method (8, 9). The ripples were characterized by the ratio t/D of their height t to the corrugation diameter D (inset of Fig. 1*F*). Corrugated supercells comprising 8×8 graphene or hBN unit cells with a nonzero t/D were created by applying biaxial compression. The initial states were constructed using H_2 being physiosorbed on the surface. Then, the H_2 molecule was allowed to undergo dissociation until a final state was reached in which two hydrogen adatoms were chemisorbed at specific locations. The electron distribution was optimized during the reaction process. The energy barrier E_b for the reaction pathway was calculated using the climbing-image nudged elastic band (NEB) method (10). All atoms were allowed to fully relax to the ground states and the spin polarization was also taken into account.

Fig. S2 shows evolution of E_b and the chemisorption energy E_c for hydrogen dissociation on graphene ripples with increasing their t/D. The final state constitutes two H adatoms being adsorbed at the central positions of the graphene hexagon (inset of Fig. 1*F*). We found this configuration to be most energetically favorable for hydrogen dissociation, after trying many different adsorption positions and reaction pathways. In agreement with the earlier report (1), the dissociation reaction becomes energetically favorable (that is, exothermic with $E_c < 0$) only for ripples with t/D larger than ~10%, and the barrier E_b also decreases to < 1 eV for such ripples. A further increase in the curvature leads to a rapid decrease in the energy barrier. For example, $E_b \approx 0.4$ eV is attained at $t/D \approx 13\%$ (Fig. S2*B*). Such nanoripples were visualized on suspended graphene membranes by high-resolution electron and tunnelling microscopies (11-13). The inferred E_b is also consistent with the reaction activation energy estimated from our experiments (Figs. 2 and 3). The above analysis supports the concept of strongly-curved nanoripples as the reactive sites for hydrogen dissociation on graphene.

Our DFT analysis for hBN monolayers revealed a radically different behavior. The central position for similar hBN ripples is energetically unfavorable ($E_c > 0$) for the whole range of t/D considered in our calculations (Fig. S2*A*). Larger curvatures (> 15%) are unrealistic for hBN because they would eventually break the crystal lattice. In addition to the central position, we have carried out DFT simulations for H atoms being adsorbed at other positions including the bridge B-N, nearest N-N and nearest B-B positions (insets of Fig. S3*C*). In all cases, we found the hydrogen splitting to be energetically unfavorable. This shows that, unlike nanorippled graphene, nanorippled hBN is highly inert, at least with respect to hydrogen dissociation. In addition, we analyzed the effect of pure strain on dissociation of H_2 by considering biaxial tensile strain for a flat hBN lattice (Fig. S3). Same as in the case of hBN ripples, the reaction was found energetically unfavorable ($E_c > 0$) for all positions and reasonable strains considered (Fig. S3).

4. Different reactivity of graphene and hBN, according to DFT analysis. The discussed splitting of H_2 on graphene and hBN surfaces involves the breaking of H-H bonds in the dissociated molecule, resulting in H atoms, and their bonding to the crystal lattice to form C-H or B/N-H bonds. Accordingly, the energy evolution of this reaction should be determined by the lengths and strengths of the involved bonds. Trying to understand the different reactivities of graphene and hBN ripples, we performed two additional calculations.

First, we analyzed the evolution of bond lengths (namely, H-H bonds and C-H or B/N-H bonds) along the hydrogen dissociation pathway. Fig. S4 shows the potential energy surface (PES) for graphene and hBN ripples as a function of the bond length between two dissociated hydrogen atoms ($d_{H\text{-}H}$) and their distance to the lattice ($d_{H\text{-}C}$ or $d_{H\text{-}B/N}$). According to the shape of the saddle points, the transition states (TS) identify the evolution of the pathway profiles from the initial states

(IS) to the final states (FS). By comparing the bond lengths at TS, we have found that dissociation of H_2 on graphene ripples requires a shorter elongation of H-H bonds than that on hBN ripples and, simultaneously, the former reaction can happen at a longer distance from the surface. Therefore, the energy cost for the reaction of H_2 with graphene ripples should be notably smaller than that with hBN ripples, as clearly revealed by the PES.

Second, we analyzed bonding strengths of atomic hydrogen to graphene and hBN ripples. To this end, we used the crystal orbital Hamilton population (COHP) method (14). As shown in Fig. S5, the negative and positive COHP values correspond to the antibonding and bonding states, respectively. For graphene ripples (Fig. S5*A*), the occupied states are all bonding states with energy distributed between -10 eV and -5 eV, below the Fermi level (E_f) whereas the unoccupied states are all antibonding and located at least 3 eV above E_f. This configuration indicates a stable adsorption of atomic H on graphene ripples. In contrast, hBN ripples exhibit a notable antibonding component in the occupied states at energies between 0 and -3 eV (Fig. S5*B*). Accordingly, the total energy is increased, resulting in a less stable adsorption configuration. This analysis provides an additional understanding of the difference between reactivities of graphene and hBN ripples.

5. Raman measurements. Our monolayer graphene crystals were either exfoliated onto an oxidized silicon wafer (RMS ≈ 0.5 nm) or transferred onto an atomically flat surface of graphite (Fig. S6). In the latter case, crystallographic axes of graphene and graphite were intentionally non-aligned. We tried other atomically flat surfaces obtained by exfoliation of layered materials but all of them exhibited a large background in the spectral region of graphene's D peak.

For Raman analysis, graphene samples were placed into a vacuum-tight heating stage with a quartz optical window (*Linkam*). The stage was employed in combination with Raman microscope *WITec*. During the measurements, a continuous flow of either hydrogen or helium was provided into the stage through dedicated gas in- and out- lets. If repeated measurements were necessary to acquire temperature dependences or check the effect of different gases, Raman spectra were acquired for the same spots far away from graphene edges.

To illustrate how annealing in a helium atmosphere influenced Raman spectra of hydrogenated graphene, we provide Fig. S7. This is in addition to the data points in the inset of Fig. 2*B*. The D peak developed due to heat-treatment of graphene in hydrogen was clearly reduced after its exposure to the same temperature but in helium. Annealing for longer times in either helium or vacuum led to a further decrease in the D peak amplitude but a residual hump in the D peak region remained even after several days. This can be attributed to the formation of stable regions of graphane, in agreement with the previous report (15).

6. Details of hydrogen isotope exchange reaction. Monolayer graphene powder (*ACS Material*, specific surface area ~1000 m^2 g^{-1}) was used as a catalyst for dissociation-recombination reaction $H_2 + D_2 \leftrightarrow 2HD$ as described in the main text. Detailed characterization of the powder is provided in ref. (16). For comparison, we tested other carbon-based materials as possible catalysts, namely, few-layer graphene obtained by ultrasonic exfoliation (17) (typical thickness of 10 layers; specific surface area of 100–200 m^2 g^{-1}) and activated charcoal (*J. L. BRAGG'S*, specific surface area of ~1000 m^2 g^{-1}). Fig. S8 shows morphologies of the three carbon-based materials. As a reference, we also employed the known catalysts for the HD reaction (18, 19): ZrO_2 (particle size, ~5 μm; specific surface area, ~0.2 m^2 g^{-1}), MgO (particle size, ≤ 50 nm; specific surface area, ~30 m^2 g^{-1}) and Cu (particle size, ~50 μm, specific surface area, ~0.01 m^2 g^{-1}). The latter materials were acquired from *Sigma-Aldrich*.

To assess their catalytic efficiency, the entire volume of a quartz tube (length of 300 mm; inner diameter of 5 mm) was filled up with one of the tested materials. The powders were loosely packed allowing easy access of gases throughout the tube and its vacuumization. Because of large differences in densities and packing, the weight of the powders placed inside the tube varied considerably. To be specific, it required ~10 mg of the monolayer graphene powder, ~200 mg of the few-layer one, ~1 g of activated charcoal and 2 to 10 g of the ZrO_2, MgO and Cu powders. The tube was then sealed and pumped down to ~10^{-3} mbar. A 50% H_2 – 50% D_2 mixture with the total pressure $P \approx 1$ bar was put into the chamber and heat-treated at a chosen temperature (up to 600 °C) for a specific length of time.

For mass spectrometry measurements, the heat-treated gas was allowed to flow through an aperture of the known diameter (typically, 6 μm). The aperture was made within a freestanding silicon-nitride membrane (500 nm thick). HD flow rates at different feed pressures were measured by a leak detector that allowed measurements for masses 2, 3 and 4 (*Leybold*). The spectrometer was calibrated using commercially supplied HD, D_2 and H_2 gases (*Sigma-Aldrich*). Fig. S9*A* shows examples of our measurements after heat-treating the H_2 – D_2 mixture with various catalysts under the same conditions (600 °C for 5 h). From these curves, the saturation HD concentrations ρ_{HD} could be calculated as done in Fig. 3*B* of the main text.

We have also measured the HD production as a function of time for the reference catalysts. They exhibited kinetic behaviors similar to that of the monolayer graphene powder (Fig. S9*B*). The saturation in HD production was typically observed after 1 h at 600 °C for ZrO_2 and MgO, whereas it required ~5 h for Cu, similar to the case of graphene. Figs. S9*C*-*D* compare the total numbers of HD molecules, N_{HD}, produced using the different standard catalysts and carbon-based materials. The N_{HD} values were normalized with respect to both weight and surface area.

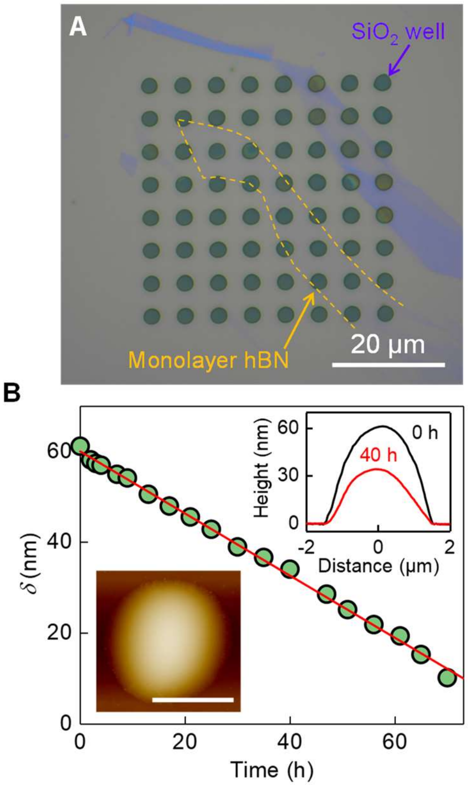

Fig. S1. Silicon-oxide microcontainers. (*A*) Optical micrograph of an array of microwells etched in silicon oxide and sealed with monolayer hBN. The dashed curve outlines the monolayer position. The blueish regions are thicker hBN. (*B*) Deflation for a representative SiO_2 microcontainer after its storage under 2 bar of Ar for 10 days. Lower inset: AFM image after the pressurization. Color scale (dark-to-bright), 0 to 60 nm. Scale bar, 2 μm. Upper inset: height profiles acquired within minutes after the microcontainer was taken out of a pressure chamber and after 40 hours (color coded). All the AFM measurements were done in air at room temperature.

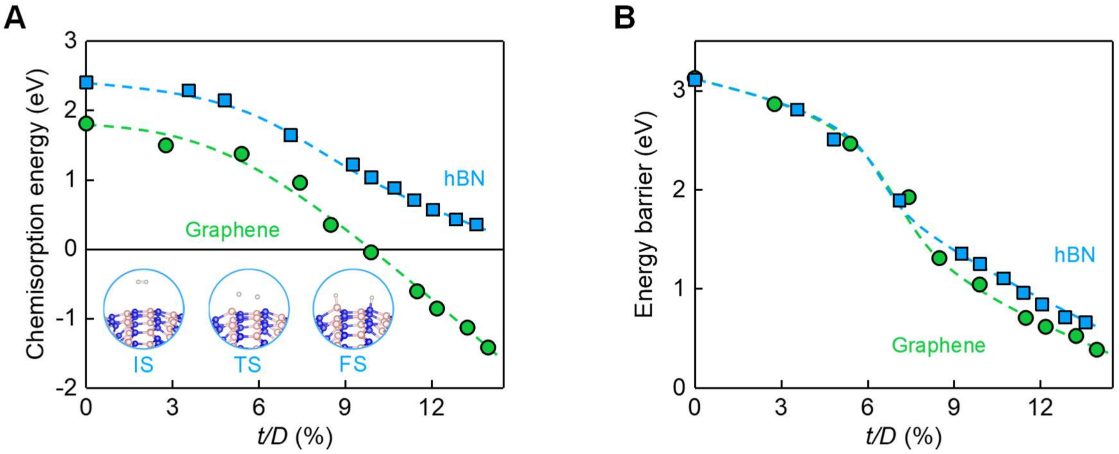

Fig. S2. Hydrogen dissociation at graphene and hBN ripples. (*A*) Chemisorption energy E_c and (*B*) energy barrier E_b as a function of *t/D* for graphene and hBN (color coded). The hydrogen adatoms are adsorbed at the central positions illustrated in the insets of Fig. 1*F*. Insets in (*A*): schematics of the initial (IS), transitional (TS) and final states (FS) for this process in the case of hBN ripples. Dashed curves: guides to the eye.

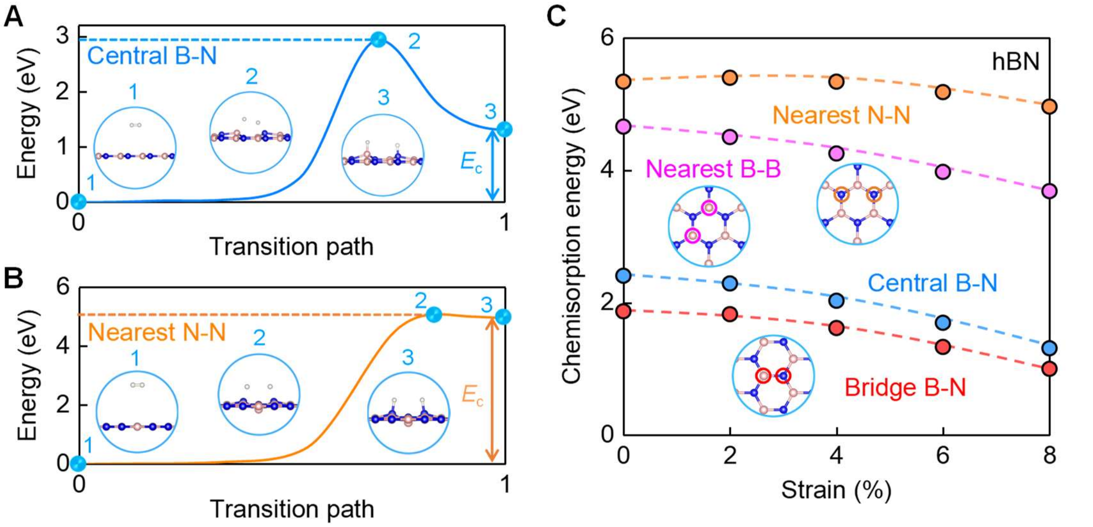

Fig. S3. Hydrogen dissociation on hBN under strain. Energy profiles for hydrogen dissociation on hBN with two H adatoms being adsorbed at (*A*) central B-N and (*B*) nearest N-N positions. A biaxial tensile strain of 8% is applied to the flat hBN lattice. Insets in (*A*, *B*) schematically show the atomic configurations of IS, TS and FS, respectively. (*C*) Chemisorption energy E_c versus strain for different adsorption positions (color coded and illustrated in the insets). Dashed curves: guides to the eye.

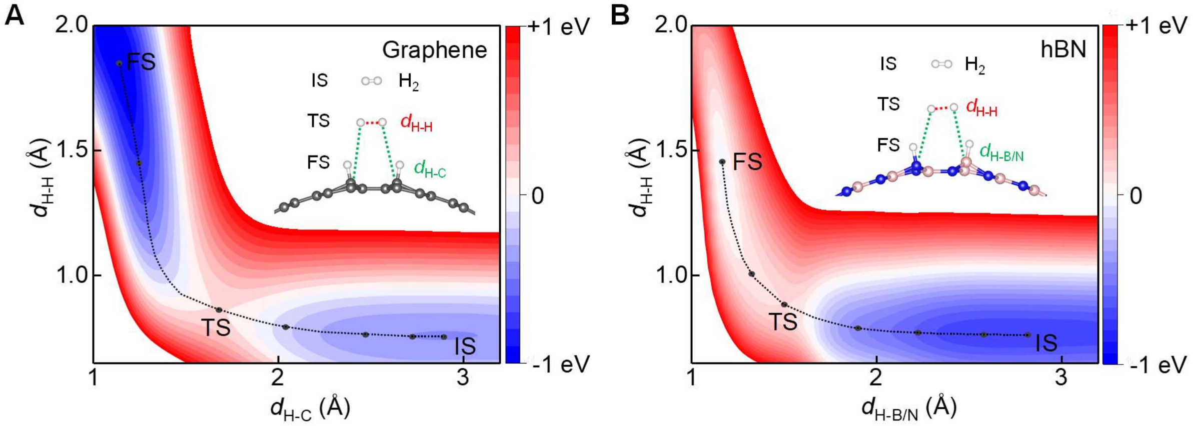

Fig. S4. Potential energy surface (PES) and changes in bond lengths. The PES for hydrogen dissociation at the central position for (*A*) graphene and (*B*) hBN ripples as a function of the distance between two dissociated H atoms (d_{H-H}), and their distance to the lattice (d_{H-C} or $d_{H-B/N}$). In both cases, t/D = 12%. The black dashed curves indicate the minimum energy paths. Insets: schematics of the dissociation process.

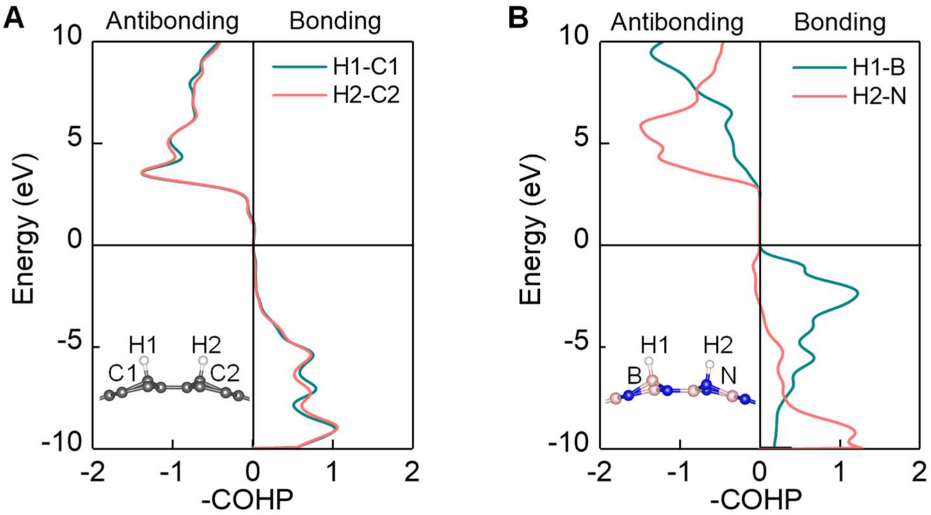

Fig. S5. Crystal orbital Hamilton population (COHP) bonding analysis. Interaction of hydrogen adatoms with (*A*) graphene and (*B*) hBN ripples (t/D = 12%). Insets: illustration of atomic structures for hydrogen adatoms adsorbed at the central position for graphene and hBN ripples.

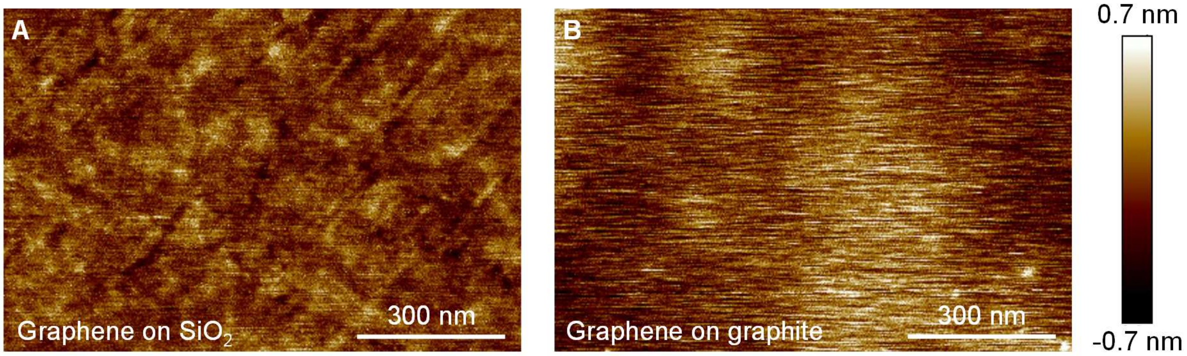

Fig. S6. AFM images of graphene on different substrates. Exfoliated monolayer crystals were deposited onto (*A*) a silicon oxide wafer and (*B*) an atomically flat surface of graphite. Same color scale (-0.7 nm to +0.7 nm) for both images. Nanoscale local corrugations are evident for graphene on SiO_2 but indiscernible above noise for graphene on graphite (only smooth variations in height are visible in the latter case).

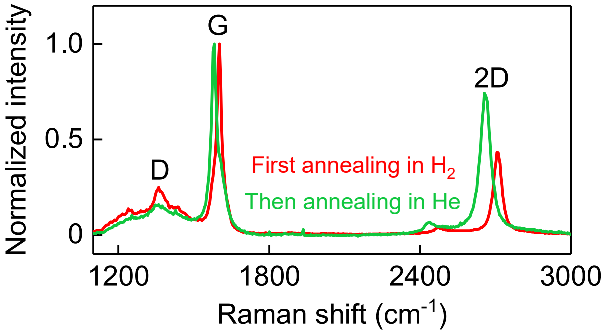

Fig. S7. Changes in Raman spectra of hydrogenated graphene after its annealing in helium. The spectra were taken at room *T* from the same position and normalized to have the same amplitude of the G peak. Red curve: after the initial exposure to 1-bar hydrogen at 600 °C for 2 hours. The developed D peak could be partially annealed using a helium atmosphere (green curve; 600 °C for 8 h). Same laser parameters as specified in Fig. 2 of the main text.

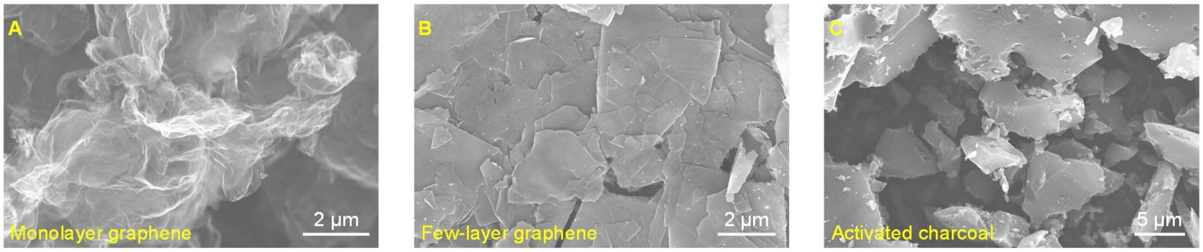

Fig. S8. Electron micrographs of the three carbon-based materials tested as catalysts. (*A*) Monolayer graphene powder. (*B*) Few-layer graphene powder. (*C*) Activated charcoal.

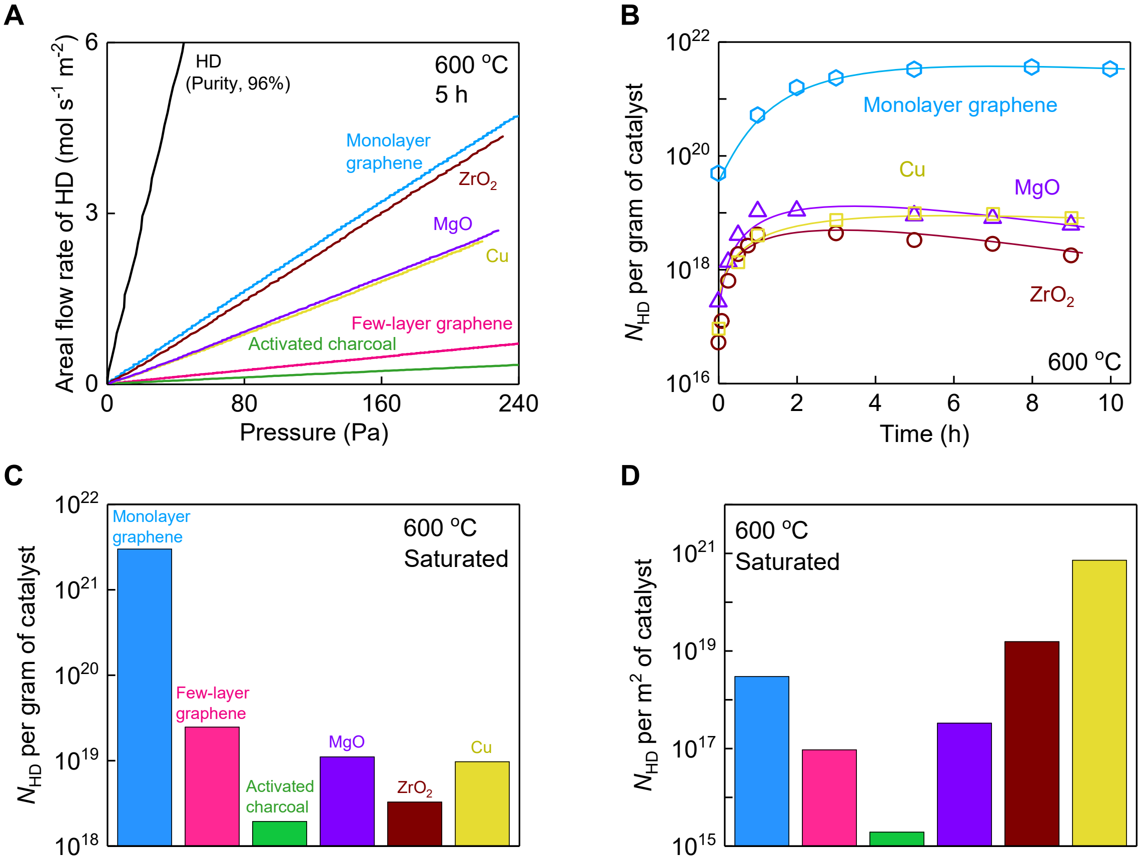

Fig. S9. Determining HD in the heat-treated hydrogen-deuterium mixtures. (*A*) Flow rates of HD through a micrometer aperture as a function of the feed pressure. In this case, a 50% H_2 – 50% D_2 mixture was annealed at 600 °C for 5 h using different catalysts (color coded). (*B*) HD production as a function of time for ZrO_2, MgO, Cu and graphene (color coded). Solid curves: guides to the eye. (*C*) Total amount of the produced HD (N_{HD} per gram) after the saturation was reached for all the tested materials (5 h at 600 °C). (*D*) Same as in panel (*C*) but recalculated in terms of the surface area.

SI References

1. P. Z. Sun *et al.*, Limits on gas impermeability of graphene. *Nature* **579**, 229–232 (2020).
2. P. Z. Sun *et al.*, Exponentially selective molecular sieving through angstrom pores. *Nat. Commun.* **12**, 7170 (2021).
3. J. S. Bunch *et al.*, Impermeable atomic membranes from graphene sheets. *Nano Lett.* **8**, 2458–2462 (2008).
4. S. P. Koenig, L. Wang, J. Pellegrino, J. S. Bunch, Selective molecular sieving through porous graphene. *Nat. Nanotechnol.* **7**, 728–732 (2012).
5. G. Kresse, J. Furthmuller, Efficient iterative schemes for ab initio total-energy calculations using a plane-wave basis set. *Phys. Rev. B* **54**, 11169–11186 (1996).
6. J. P Perdew, K. Burke, M. M. Ernzerhof, Generalized gradient approximation made simple. *Phys. Rev. Lett.* **77**, 3865–3868 (1996).
7. H. J. Monkhorst, J. D. Pack, Special points for Brillouin-zone integrations. *Phys. Rev. B* **13**, 5188–5192 (1976).
8. S. Grimme, Semiempirical GGA-type density functional constructed with a long-range dispersion correction. *J. Comput. Chem.* **27**, 1787–1799 (2006).
9. T. Kerber, M. Sierka, J. Sauer, Application of semiempirical long-range dispersion corrections to periodic systems in density functional theory. *J. Comput. Chem.* **29**, 2088–2097 (2008).
10. D. Sheppard, P. Xiao, W. Chemelewski, D. D. Johnson, G. Henkelman, A generalized solid-state nudged elastic band method. *J. Chem. Phys.* **136**, 074103 (2012).
11. J. C. Meyer *et al.*, On the roughness of single- and bi-layer graphene membranes. *Solid State Commun.* **143**, 101–109 (2007).
12. R. Zan *et al.*, Scanning tunnelling microscopy of suspended graphene. *Nanoscale* **4**, 3065–3068 (2012).
13. P. Xu *et al.*, Unusual ultra-low-frequency fluctuations in freestanding graphene. *Nat. Commun.* **5**, 3720 (2014).
14. V. L. Deringer, A. L. Tchougréeff, R. Dronskowski, Crystal orbital Hamilton population (COHP) analysis as projected from plane-wave basis sets. *J. Phys. Chem. A* **115**, 5461–5466 (2011).
15. D. C. Elias *et al.*, Control of graphene's properties by reversible hydrogenation: evidence for graphane. *Science* **323**, 610–613 (2009).
16. J. A. Hondred, L. R. Stromberg, C. L. Mosher, J. C. Claussen, High-resolution graphene films for electrochemical sensing via inkjet maskless lithography. *ACS Nano* **11**, 9836–9845 (2017).
17. Y. Hernandez *et al.*, High-yield production of graphene by liquid-phase exfoliation of graphite. *Nat. Nanotechnol.* **3**, 563–568 (2008).
18. G. C. Bond, Catalysis by Metals (Academic, New York, 1962).
19. D. A. Dowden, N. Mackenzie, B. M. V. Trapnell, Hydrogen-deuterium exchange on the oxides of transition metals. *Adv. Catal.* **9**, 65–69 (1957).
